# Membrane Adsorption Enhances Translocation of Antimicrobial Peptide Buforin 2

**DOI:** 10.1021/acs.jpcb.4c04338

**Published:** 2024-08-28

**Authors:** Mehrnoosh Khodam Hazrati, Robert Vácha

**Affiliations:** † CEITEC − Central European Institute of Technology, Masaryk University, Kamenice 753/5, Brno 625 00, Czech Republic; ‡ National Centre for Biomolecular Research, Faculty of Science, Masaryk University, Kamenice 5, Brno 625 00, Czech Republic; § Department of Condensed Matter Physics, Faculty of Science, Masaryk University, Kotlářská 267/2, Brno 611 37, Czech Republic

## Abstract

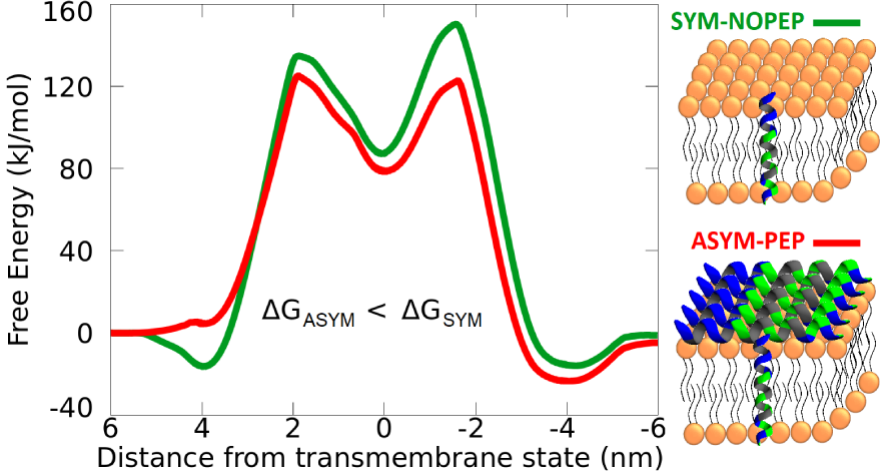

Despite ongoing research on antimicrobial peptides (AMPs) and cell-penetrating peptides (CPPs), their precise translocation mechanism remains elusive. This includes Buforin 2 (BF2), a well-known AMP, for which spontaneous translocation across the membrane has been proposed but a high barrier has been calculated. Here, we used computer simulations to investigate the effect of a nonequilibrium situation where the peptides are adsorbed on one side of the lipid bilayer, mimicking experimental conditions. We demonstrated that the asymmetric membrane adsorption of BF2 enhances its translocation across the lipid bilayer by lowering the energy barrier by tens of kJ mol^–1^. We showed that asymmetric membrane adsorption also reduced the free energy barrier of lipid flip-flop but remained unlikely even at BF2 surface saturation. These results provide insight into the driving forces behind membrane translocation of cell-penetrating peptides in nonequilibrium conditions, mimicking experiments.

## Introduction

Membrane translocation is a critical step in the molecular mechanism of cell-penetrating peptides (CPPs) and some antimicrobial peptides (AMPs) that have intracellular targets.^1^ Various mechanisms have been proposed to explain the translocation of CPPs which can be broadly categorized as either energy-independent penetration or energy-dependent mechanisms such as endocytosis and macropinocytosis.^2^ Elucidation of the precise translocation mechanism is essential not only for our fundamental understanding of these peptides but also for the rational design of synthetic analogs with improved penetrating properties.^3^

Among antimicrobial peptides (AMPs), BF2 has received considerable attention due to its potent antimicrobial activity against a wide spectrum of pathogenic bacteria.^4−7^ BF2 is a 21-amino acid AMP (Sequence: TRSSRAGLQFPVGRVHRLLRK) that penetrates inside the cell and accumulates in the cytoplasm leading to bacterial death without triggering membrane lysis.^8,9^ The typical length of α-helical BF2 is approximately 3 nm, with a diameter of around 1 nm. BF2 membrane translocation is known to be independent of cellular receptors because it can readily enter various bacterial cells as well as vesicles that contain only lipids in their membrane.^10^ It has previously been suggested that the presence of the proline residue in the BF2 sequence is responsible for its spontaneous cell-penetrating ability by providing flexibility to the peptide.^11,12^ However, the free energy barrier for BF2 translocation across a symmetric membrane indicates a nonspontaneous process, regardless of the presence of proline.^13^ The translocation mechanism of BF2 thus remains enigmatic, with several competing hypotheses proposed in the literature.^14,15^

In this study, we used coarse-grained (CG) and all-atom (AA) molecular dynamics simulations to investigate the details of the BF2 translocation process across a model bacterial membrane. Similar to experiments where peptides interact with cells from one side, we constructed a lipid bilayer with peptides adsorbed on one leaflet and calculated the effect on the translocation barrier of BF2. In addition to peptide translocation, we also evaluated the change in lipid flip-flop. We hypothesize that peptide adsorption will modify the translocation barrier because Lee previously showed that the barrier to pore formation is reduced when peptides are distributed on one side of the membrane.^16^ In addition, the asymmetric composition of lipid membranes has been demonstrated to affect the translocation of molecules.^17^

## Methods

### Coarse-Grained Simulations

All coarse-grained (CG) molecular dynamics simulations were performed using Gromacs versions 2020.3 and 2021.4^18,19^ with Martini v3.0.0 force field.^20^

The initial configuration of the membrane was generated using the insane.py script.^21^ The bilayer was formed by 512 lipids, 384 1-palmitoyl-2-oleoyl-phosphatidyl-ethanolamine (POPE), and 128 1-palmitoyl-2-oleoyl-*sn*-glycero-3-phosphoglycerol (POPG) (3:1 mol:mol) as a simple mimic of Gram-negative bacterial membranes.^22^ Lipids were equally distributed in both leaflets. The system was solvated with approximately 35 water beads per lipid and Na^+^ and Cl^–^ ions at physiological concentrations of 150 mM. Excess Na^+^ was used to balance the net charge on the system. BF2 was modeled at a physiological pH of 7.4, where it assumed a net charge of ^+^6. This protonation state is due to the presence of multiple lysine and arginine residues, which are positively charged under these conditions. The possible change of the protonation state upon the peptide insertion into the membrane was not evaluated and remained the same in all our simulations.

Martinize.py script^23^ was applied to convert the BF2 atomic structures to the Martini CG structure and topology. To investigate the effect of peptide crowding on one side of the membrane, we studied four different systems: 1) a symmetric POPE:POPG (3:1 mol:mol) membrane with only translocating peptide and no additional adsorbed peptides (hereafter “SYM-NOPEP”), 2) a peptide-asymmetric POPE:POPG (3:1 mol:mol) membrane containing 20 BF2 on one leaflet (“ASYM-PEP”), 3) a symmetric POPE:POPG (3:1 mol:mol) membrane with 20 BF2 on each leaflet (“SYM-PEP”), and 4) an asymmetric POPE:POPG (3:1 mol:mol) membrane with 20 BF2 on one leaflet and additional area-matching lipids in the opposite leaflet (ASYM-LIP). To make the latter, we added extra lipids (21 POPE and 7 POPG molecules) to the leaflet without any peptides to reach the same area as the other leaflet, which contains peptides. The BF2 peptides retained adsorbed on the leaflets during the simulation. To keep the number of adsorbed peptides on the membrane the same, i.e., preventing desorption, we used flat-bottomed potentials on each peptide with 100 kJ mol^–1^ force constant in the Z direction of the membrane’s local center of mass.

Furthermore, in all systems, a BF2 was positioned in the phosphate region at one of the membrane leaflets with its hydrophobic residues facing the acyl tails in order to perform the calculation of the free energy associated with the translocation process.

The systems were minimized and then equilibrated for at least 100 ns by using the NpT ensemble with a time step of 20 fs. The Parrinello–Rahman barostat^24,25^ with a semi-isotropic scheme and a coupling constant of 12 ps was used to maintain the pressure at 1 bar. The temperature was maintained at 310 K using the velocity-rescaling thermostat modified with a stochastic term^26^ with a coupling constant of 1.0 ps. The relative dielectric constant was set to 15. At 1.1 nm, the vdW potential was shifted to 0, and all nonbonded interactions were cut off.

**Figure 1:**
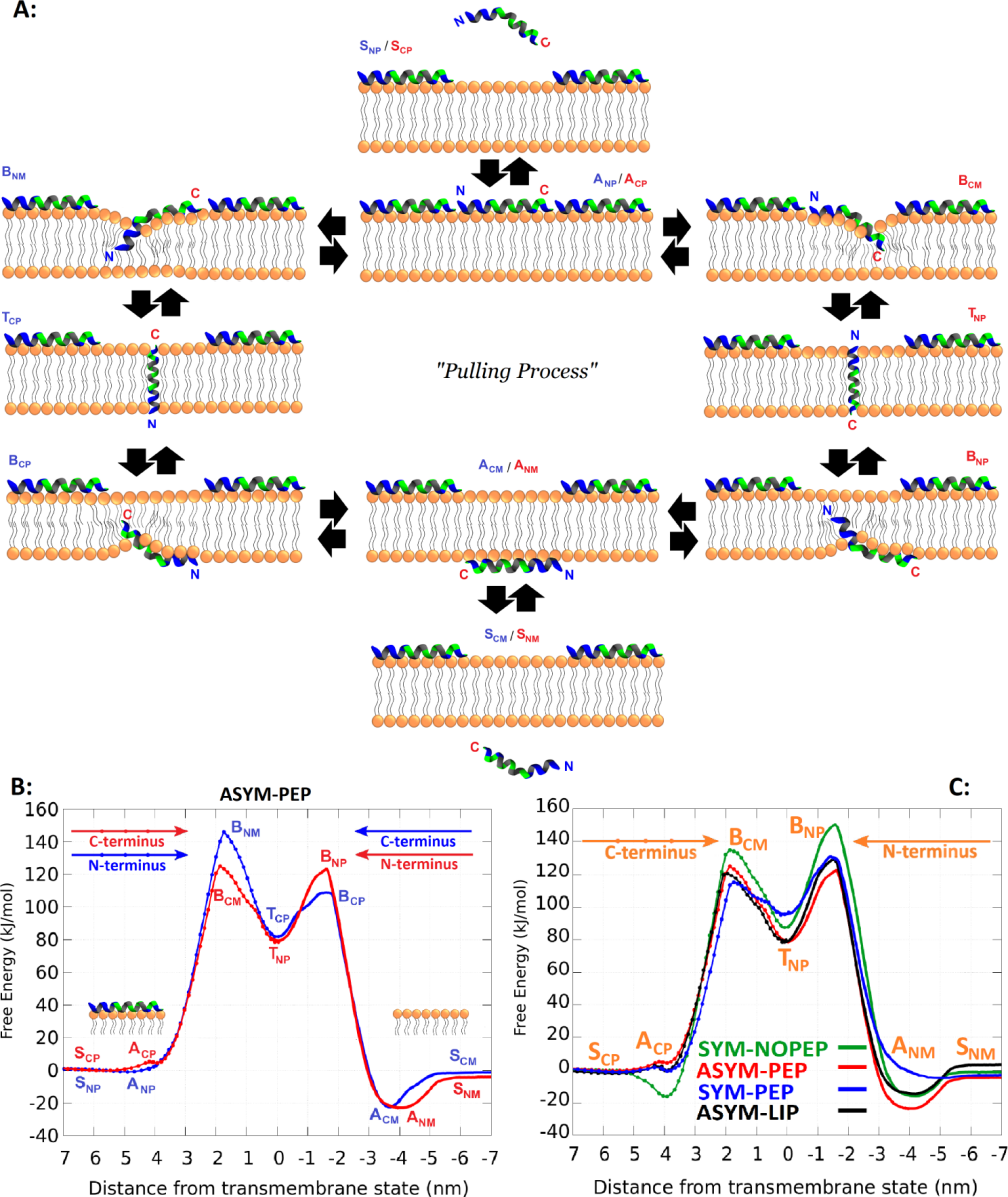
Calculation of the Buforin 2 (BF2) translocation. A: Schematic illustration of translocation pathways including BF2 adsorption and insertion starting with its *N*- or C-terminus. A symmetric membrane with BF2s adsorbed on one side was employed (ASYM-PEP). B: Calculated free energy profiles for translocating the peptide across the peptide-asymmetric membrane (ASYM-PEP) start with C- or N-terminus. C: Calculated free energy profiles for translocating the peptide via the C-terminus, which has a lower barrier than the N-terminus across the symmetric membrane without adsorbed peptides (SYM-NOPEP), the peptide-asymmetric membrane with peptides on one side (ASYM-PEP), the symmetric membrane with peptides on both sides (SYM-PEP), and the asymmetric membrane with peptides adsorbed on one leaflet and additional area-matching lipids in the opposite leaflet (ASYM-LIP). The estimated error of the free energy profiles is less than 5 kJ mol^–1^ based on the hysteresis and difference for peptide being in solution on both sides of the membrane.

To calculate the free energy of the BF2 translocation, we pulled a single peptide *N*- or C-terminus from the phosphate region on the membrane surface into the solution (adsorption) or into the membrane (insertion). Due to the asymmetric nature of the peptide, the translocation process was carried out, starting with the insertion of the N-terminus and the C-terminus separately. For example, Figure 1A shows the pulling process for the ASYM-PEP system. The peptide was pulled using the local center of mass defined with the three terminal backbone beads and the final beads of charged residues on the pulled half of the peptide. This collective variable has been shown to be suitable for peptide translocation without hysteresis.^7^

Each peptide terminus was pulled for 1 μs with a 5.00 nm μs^–1^ pulling rate and a 1000 kJ mol^–1^ nm^–2^ harmonic potential force constant. The pulling was performed in the Z direction against the local center of mass of the membrane with a cylinder radius of 2.0 nm. The initial configurations for the umbrella sampling simulations were then generated from the respective pulling trajectory. Subsequently, 83 windows were equally spaced by 0.05 nm near the water-membrane interface and by 0.10 nm in bulk water for the adsorption. Similarly, 75 equally spaced configurations by 0.04 nm near the bilayer center and by 0.08 nm in the rest of the distance range were generated for the insertion. The distances and the biased force constants used for umbrella sampling are listed in equations S1 and S2 for the adsorption and insertion processes, respectively. Free energy profiles were then calculated by sampling each window for 1–7 μs. The weighted histogram analysis (WHAM)^27^ was then used to reconstruct the free energy profiles. Figure 1B shows the free energy profiles for BF2 translocation across the ASYM-PEP membrane starting with the insertion of N- or C-terminus. Also, we compared the free energy profiles for different systems in Figure 1C. The free energy values of important states for Figure 1B,C are listed in Tables 1 and 2, respectively.

**Table 1:** Free Energy Values in kJ mol^–1^ for Important States on the Translocation Free Energy Profiles in Figure 1B.^a^

S_*NP*_	A_*NP*_	B_*NM*_	T_*CP*_	B_*CP*_	A_*CM*_	S_*CM*_
0	0	146	82	108	–21	–1
S_*CP*_	A_*CP*_	B_*CM*_	T_*NP*_	B_*NP*_	A_*NM*_	S_*NM*_
0	6	124	79	123	–23	–4

a The error was estimated to be below 5 kJ mol^–1^

**Table 2:** Free Energy Values in kJ mol^–1^ for Important States on the Translocation Free Energy Profiles in Figure 1C.^a^

	S_*CP*_	A_*CP*_	B_*CM*_	T_*NP*_	B_*NP*_	A_*NM*_	S_*NM*_
SYM-NOPEP	0	–16	133	88	151	–15	0
ASYM-PEP	0	6	124	79	123	–23	–4
SYM-PEP	0	–2	113	96	131	2	–2
ASYM-LIP	0	–2	122	79	129	–15	4

a The error was estimated below 5 kJ mol^–1^

**Figure 2:**
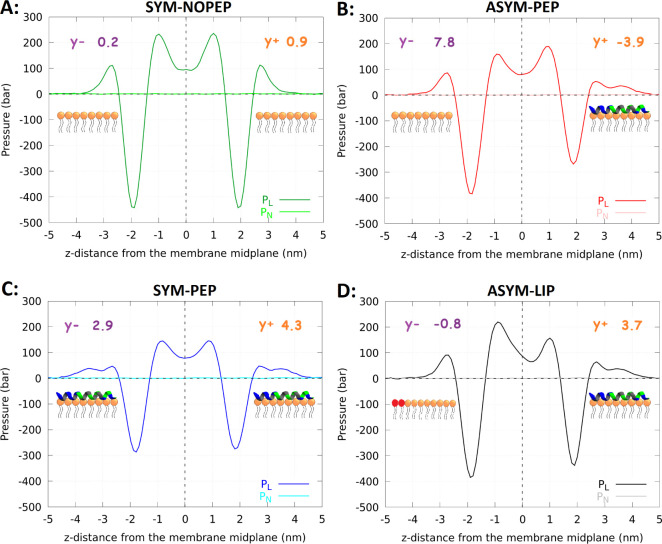
Lateral (P_*L*_) and normal (P_*N*_) pressure profiles along the membrane normal. The tensions of the upper (Y_+_, orange) and lower (Y_–_, purple) leaflets are presented in units of *mN/m*. Based on the SYM-NOPEP and SYM-PEP systems, the tension error was estimated to be approximately 2.5 mN/m.

Furthermore, we calculated the pressure profiles and tension of the upper and lower leaflets in all systems, including the SYM-NOPEP, ASYM-PEP, SYM-PEP, and ASYM-LIP systems. The pressure profiles were calculated by the Gromacs-LS package v2016.3^28^ and are shown in Figure 2A–D. For details concerning the calculation procedure, refer to the Bartos et. al article.^17^ We used the code *Order* to calculate the CG lipid order parameters. Further details regarding the calculation of order parameters can be found in the Supporting Information.

We also calculated the free energy of the flip-flop of an individual POPE lipid for the SYM-NOPEP and ASYM-PEP systems. We pulled a phosphate group from the upper leaflet to the lower leaflet and vice versa. In the SYM-NOPEP system, we only pulled the phosphate group from the upper leaflet to the lower leaflet due to the system symmetry. The phosphates were pulled for 1 μs with a 5.00 nm μs^–1^ pulling rate and a 5000 kJ mol^–1^ nm^–2^ harmonic potential force constant. Consequently, 67 equally spaced configurations by 0.05 nm near the bilayer center and by 0.10 nm in the rest of the distance range were extracted for the umbrella sampling, and free energy profiles were then calculated by sampling each window for 1 μs. The distances and the biased force constants used for flip-flop umbrella sampling are listed in equation S3. The weighted histogram analysis (WHAM)^27^ was then used to reconstruct the free energy profiles. We used the windows of both pulling directions for calculating the free energy profile of the ASYM-PEP system.

### All-Atom Simulations

To validate our CG results, we performed all-atom (AA) simulations and calculated the free energy of the flip-flop (translocation) of an individual POPE lipid in the SYM-NOPEP and ASYM-PEP systems. All simulations were performed using the CHARMM36 force field^29^ and the same version of Gromacs as CG simulations.^18,19^

The initial POPE:POPG (3:1 mol:mol) membrane was assembled with the Z axis as the membrane normal using the Bilayer Builder tool in the CHARMM-GUI web server.^30,31^ The membranes were formed with 512 lipids equally distributed in both leaflets. BF2 peptides were produced by AmberTools18.^32^ In the ASYM-PEP system, the peptides were placed and kept in the headgroups region at one of the membrane leaflets using flat-bottomed potentials applied on each peptide with 100 kJ mol^–1^ force constant in the Z direction of the membrane’s local center of mass. The system was then solvated with approximately 50 water molecules per lipid. Na^+^ and Cl^–^ ions were added to the system at physiological concentrations of 150 mM, and extra Na^+^ was used to balance the net charge on the system.

The simulations were performed in the NpT ensemble with a constant temperature and pressure using a time step of 2 fs. A 1.0–1.2 nm force-switching scheme was used to evaluate the vdW forces, and short-range electrostatic interactions were cut off at 1.2 nm. Particle Mesh Ewald (PME) method^33^ was used for treating the long-range electrostatic interactions using PME order 4 and a cutoff of 1.2 nm. The systems were initially minimized and equilibrated for 200 ns at 310 K and 1 bar using the Berendsen thermostat and barostat with a coupling constant of 1.0 and 5.0 ps, respectively.^34^ After equilibration, the P atom of a POPE lipid was pulled for 100 ns with a 50 nm μs^–1^ pulling rate and a 5000 kJ mol^–1^ nm^–2^ harmonic potential force constant. We pulled a phosphate group from the upper leaflet to the lower leaflet and vice versa for both SYM-NOPEP and ASYM-PEP systems. During pulling, the temperature was kept at 310 K using the Nose-hoover thermostat^35^ with a time constant of 1.0 ps applied separately for membrane, solvent, and peptides when present. Semi-isotropic pressure of 1 bar was applied independently in the membrane plane and along the membrane normal using the Parrinello–Rahman algorithm^24^ with a coupling constant of 10.0 ps and compressibility of 4.5e-5 bar^–1^. In total, 91 equally spaced configurations by 0.01 nm near the bilayer center and by 0.05–0.10 nm in the rest of the distance were used for the umbrella sampling. The distances and the biased force constants used for flip-flop umbrella sampling are listed in equation S3. Free energy profiles were calculated from each window sampled for 400 ns and reconstructed using the weighted histogram analysis (WHAM) method^27^ and the windows of both pulling directions.

## Results and Discussion

To evaluate how the asymmetric adsorption of peptides on the membrane affects peptide translocation, we performed coarse-grained simulations. We calculated the free energy profiles consisting of BF2 adsorption on the membrane surface and BF2 insertion into the membrane. We determined the barriers to translocation and the factors influencing the free energy profiles.

Figure 1A shows the adsorption and insertion paths starting with the N- or C-terminus of the BF2 peptide into the asymmetric system composed of POPE:POPG (3:1 mol:mol) membrane with peptides on one side (ASYM-PEP). The pulling process for the ASYM-PEP system was carried out in eight subprocesses, which were combined to obtain the full translocation free energy profile: [1] the adsorption of the N-terminus and C-terminus onto both upper and lower leaflets and [2] the insertion of the N-terminus and C-terminus into the upper and lower leaflets. In the case of the symmetric membrane, half of the processes are the same because there is no difference between the membrane leaflets.

Figure 1B shows the free energy profiles for BF2 translocation across the ASYM-PEP membrane starting with the insertion of N- or C-terminus. The profiles show that the free energy barrier is lower by about 10 kJ mol^–1^ when the insertion into the leaflet covered with peptides starts with the C-terminus rather than the N-terminus. Note that during the simulation we observed the formation of peptide dimers. However, it probably does not have a strong influence on the profile because the lifetime of these dimers was short (tens of nanoseconds), and we have not observed cotranslocation of any other peptides. We further focused on more favorable translocation starting with C-terminus insertion.

We compared the BF2 translocation in ASYM-PEP with a translocation across the peptide-free symmetric lipid membrane (SYM-NOPEP), the symmetric membrane with peptides adsorbed on both leaflets (SYM-PEP), and the asymmetric membrane with peptides adsorbed on one leaflet and additional area-matching lipids in the opposite leaflet (ASYM-LIP), see Figure 1C. In the SYM-NOPEP system, also the insertion from the C-terminus has a lower free energy barrier of translocation by about 10 kJ mol^–1^ compared to the insertion starting with the N-terminus, which is in agreement with the previous study.^13^ There is a free energy minimum at the headgroup region about 2 nm from the membrane center in the SYM-NOPEP system (see the green curve), representing favorable peptide adsorption on the membrane. The membrane adsorption of peptides in the ASYM-PEP system destabilizes the adsorbed state of additional peptides, and the minimum is lost. In the ASYM-PEP system, the transmembrane state was further stabilized by about 10 kJ mol^–1^, and the BF2 translocation barrier (difference between the maximum and the minimum on the profile) decreased by about 25 kJ mol^–1^ compared to the SYM-NOPEP membrane. This finding confirms our hypothesis regarding enhancing peptide translocation across peptide-asymmetric systems, aligning well with previous studies.^16,17^ The addition of extra lipids to the leaflet without peptides in the ASYM-LIP membrane resulted in an adsorption state on that leaflet similar to that in the SYM-NOPEP system, while the leaflet with peptides remained consistent with the ASYM-PEP system. Furthermore, the free energy of peptide insertion from the leaflet without peptides was identical to that of the SYM-PEP system, both being intermediate between the SYM-NOPEP and ASYM-PEP systems. In the SYM-PEP system, peptide adsorption on both leaflets caused instability in the transmembrane state and led to a loss of minimums in the adsorption states on both leaflets.

There are two contributions that could decrease the translocation barrier for BF2 in the ASYM-PEP system. The first contribution is the absence of the surface minima for peptide adsorption, i.e., the surface is saturated with peptides. The second contribution is inside the membrane, where the free energy profile is lower compared to that of the SYM-NOPEP system. This decrease could be influenced by the lipid tail packing and the differences in the leaflet tensions, which have been shown to affect the peptide insertion into the hydrophobic region.^17^ While we are not aware of any experimental data that can be directly compared to the calculated free energy profiles and their differences, one can imagine such an experiment, e.g., an experiment evaluating peptide permeation at different peptide concentrations.

We calculated the differential stress in all studied systems. Apart from ASYM-PEP, SYM-NOPEP, and SYM-PEP, we constructed the ASYM-LIP system in which the number of lipids was modified in such a way that both leaflets with and without peptides had the same area. The reason is that peptide adsorption on the membrane resulted in the increase of the area per lipid from 0.63 nm^2^ to 0.70 nm^2^. Figure 2A–D shows the pressure profiles and tension of each leaflet.

Peptide adsorption decreased the tension on the corresponding leaflet and increased the tension in the opposing leaflet, resulting in significant differential stress. This can be understood in terms of the increased leaflet area after peptide adsorption. Due to the applied periodic boundary conditions, both leaflets have the same area in the simulation box. Therefore, the leaflet without peptides is expanded, while the leaflet with peptides is compressed.

The addition of extra lipids to the leaflet without peptides in the ASYM-LIP membrane resulted in relaxation of this leaflet with tension close to zero. However, there is tension in the leaflet with peptides (with the opposite sign compared to the ASYM-PEP system). The additional lipids in ASYM-LIP adjusted the area per lipid but did not completely remove the tension. The tension in the leaflet with peptides is the same as that in the symmetric membrane with peptides on both sides (SYM-PEP).

**Figure 3:**
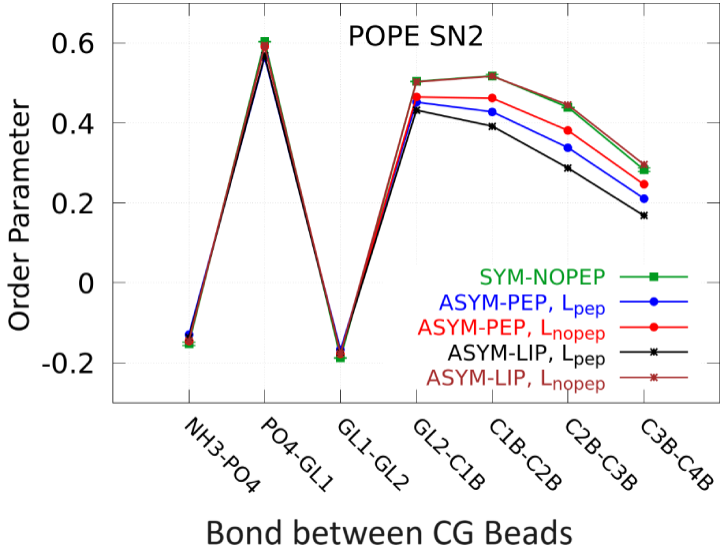
Lipid order parameter for SN2 tails of POPE from the upper and lower leaflets in the ASYM-PEP (with peptides on one of the leaflets), ASYM-LIP (the asymmetric membrane with peptides adsorbed on one leaflet and additional area-matching lipids in the opposite leaflet), and SYM-NOPEP (symmetric peptide-free) systems. L_pep_ indicates the leaflet with peptides and L_nopep_ the leaflet with no peptides in the ASYM-PEP and ASYM-LIP systems. Data for the SYM-NOPEP system are averaged over both leaflets, and error bars show the standard deviation.

Figures 3 and Figure S1 show the order parameters for SN1 and SN2 of POPE and POPG in the SYM-NOPEP, ASYM-PEP, and ASYM-LIP systems. There is a significant tail disorder in the leaflet with peptides (L_pep_)—for labels of specific bonds see Figure S4 with the CG representations of POPE and POPG lipids. Interestingly, the other leaflet (L_nopep_) in the ASYM-PEP system has also decreased the order of the lipid tails, although to a lesser extent. In the ASYM-LIP system, the leaflet without peptides (L_nopep_) has almost the same order parameter as in SYM-NOPEP, while the order in the leaflet with peptides (L_pep_) is decreased even more than in ASYM-PEP. All these results are consistent with the changes in the leaflet area, with a larger area leading to larger tail disorder. For both POPE and POPG, the disorder is more pronounced in the SN2 tails but the trends remain. The results show that the presence of peptides enhances lipid disorder in the bacterial membrane mimics and may be one of the reasons for promoting peptide translocation across the membrane. The calculated disordering of the lipid tails is in line with the previously reported decrease of the lipid order parameters in the presence of AMPs.^36^

**Figure 4:**
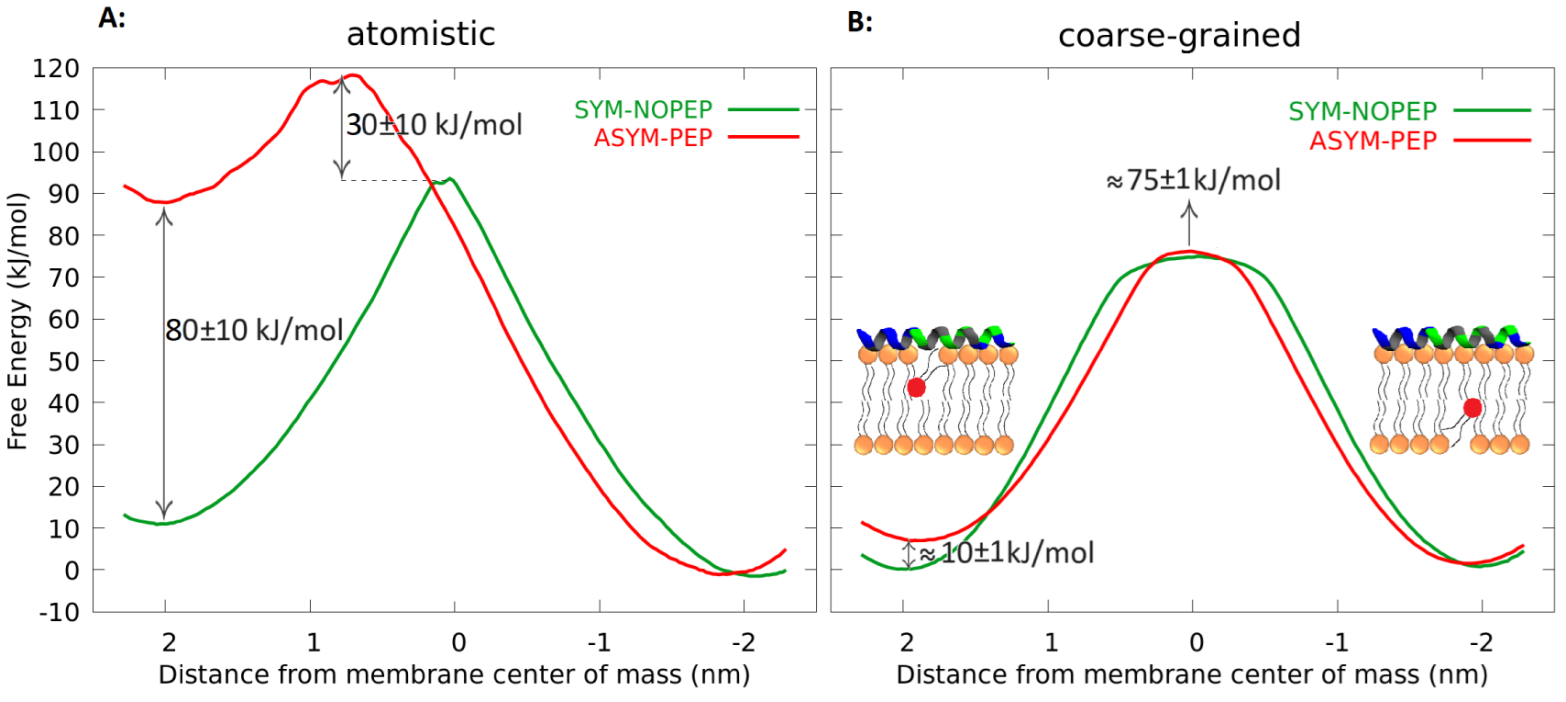
Free energy profiles for the POPE flip-flop in SYM-NOPEP (symmetric peptide-free) and ASYM-PEP (with peptides on one of the leaflets) systems obtained from A: all-atom simulations and B: coarse-grained. Free energy profiles of flip-flopping in the AA and CG systems show a hysteresis of about 10 and 1 kJ mol^–1^, respectively.

To determine whether the free energy changes are caused by specific peptide–peptide interactions or more general effects on the membrane, we also calculated the translocation free energy profiles for the lipids. Figure 4A,B show that asymmetric peptide adsorption on one leaflet decreases the free energy barrier for POPE translocation from that leaflet to the other. We confirmed this trend with all-atom simulations, where the barrier for the translocation of a POPE molecule decreased by 80 ± 10 kJ mol^–1^. In the CG simulations, no significant water defects or water channels were observed during the translocation. However, in the AA simulations, the presence of water defects related to the inserted phosphate could be the reason for the observed hysteresis.^17^ While there is agreement in the qualitative trends between the AA and CG free energy profiles, the quantitative differences are significant. These differences are likely originating from the parametrizations, but we do not have experimental data enabling their validation. The result suggests that the presence of peptides on one side of the membrane affects membrane properties such as tail order parameters and membrane tension, resulting in the facilitated translocation of both peptides and lipids.

## Conclusions

We investigated how the peptide adsorption on one side of the membrane affects the translocation of both the peptide and lipid. Using a simplified bacterial mimic membrane composed of POPE:POPG (3:1 mol:mol) and Buforin 2 (BF2) as a model peptide, we demonstrated that asymmetric peptide adsorption significantly enhances BF2 translocation. The enhanced translocation originates from the destabilization of the surface-adsorbed state of the peptide and induces the disorder of lipid tails. The asymmetric adsorption of peptides also promotes lipid translocation from the peptide-containing leaflet to another. Despite the observed significant difference between the free energy profiles for lipid flip-flops using AA and CG, the lipid flip-flop remains an improbable event even at the BF2 surface saturation in accordance with previous experimental observations. Our results shed light on the effect of asymmetric AMP adsorption on membrane permeation, suggesting that asymmetry could serve as a driving force for membrane transport.

## Supporting Information Available

Lipid order parameter for SN1 tails of POPE (Figure S1); lipid order parameter for SN1 tails of POPG (Figure S2); lipid order parameter for SN2 tails of POPG (Figure S3); POPE and POPG representations at the coarse-grained level (Figure S4) (PDF)
