# Supplementary material for: Membrane Adsorption Enhances Translocation of Antimicrobial Peptide Buforin 2
Source: J Phys Chem B. 2024 Aug 28;128(35):8469–76. doi: 10.1021/acs.jpcb.4c04338 (PMC11382259; doi:10.1021/acs.jpcb.4c04338)
Supplement: Supplementary file 1 — jp4c04338_si_001.pdf [file jp4c04338_si_001.pdf]

Supporting Information

Membrane Adsorption Enhances Translocation of Antimicrobial Peptide Buforin 2

Mehrnoosh Khodam Hazrati*,† and Robert Vácha*,†,‡,¶

† CEITEC – Central European Institute of Technology, Masaryk University, Kamenice 753/5, 625 00 Brno, Czech Republic
‡ National Centre for Biomolecular Research, Faculty of Science, Masaryk University, Kamenice 5, 625 00 Brno, Czech Republic
¶ Department of Condensed Matter Physics, Faculty of Science, Masaryk University, Kotlářská 267/2, 611 37 Brno, Czech Republic

E-mail: robert.vacha@mail.muni.cz

$$f_c, d_c = \begin{cases} 1800, 0.05 & 2.40 \leq d_{pm} \leq 5.00 \\ 1400, 0.05 & 5.05 \leq d_{pm} \leq 5.35 \\ 1000, 0.05 & 5.40 \leq d_{pm} \leq 6.00 \\ 1000, 0.10 & 6.10 \leq d_{pm} \leq 7.00 \end{cases} \tag{S1}$$

$$f_c, d_c = \begin{cases} 3000, 0.08 & -2.40 \leq d_{pm} \leq -0.40 \\ 8000, 0.04 & -0.36 \leq d_{pm} \leq 0.36 \\ 3000, 0.08 & 0.40 \leq d_{pm} \leq 2.40 \end{cases} \tag{S2}$$

Where f_c is the biased force constant (kJ/mol), d_c is the distance between configurations (nm), and d_{pm} is the oriented distance between the terminus of pulled peptide and the center of mass of the membrane (nm).

$$
f_c, d_c = \begin{cases} 1000, 0.10 & -2.30 \leq d_{pm} \leq -1.10 \\ 2000, 0.05 & -1.00 \leq d_{pm} \leq -0.30 \\ 3000, 0.05 & -0.25 \leq d_{pm} \leq -0.15 \\ 4000, 0.05 & -0.10 \leq d_{pm} \leq 0.10 \\ 3000, 0.05 & 0.15 \leq d_{pm} \leq 0.25 \\ 2000, 0.05 & 0.30 \leq d_{pm} \leq 1.00 \\ 1000, 0.10 & 1.10 \leq d_{pm} \leq 2.30 \end{cases} \tag{S3}
$$

$$
f_c, d_c = \begin{cases} 1000, 0.10 & -2.30 \leq d_{pm} \leq -1.10 \\ 2000, 0.05 & -1.00 \leq d_{pm} \leq -0.30 \\ 3000, 0.02 & -0.25 \leq d_{pm} \leq -0.12 \\ 4000, 0.01 & -0.10 \leq d_{pm} \leq 0.10 \\ 3000, 0.02 & 0.12 \leq d_{pm} \leq 0.25 \\ 2000, 0.05 & 0.30 \leq d_{pm} \leq 1.00 \\ 1000, 0.10 & 1.10 \leq d_{pm} \leq 2.30 \end{cases} \tag{S4}
$$

Where f_c is the biased force constant (kJ/mol), d_c is the distance between configurations (nm), and d_{pm} is the oriented distance between the P atom of phosphate group in the pulled lipid and the center of mass of the membrane (nm).

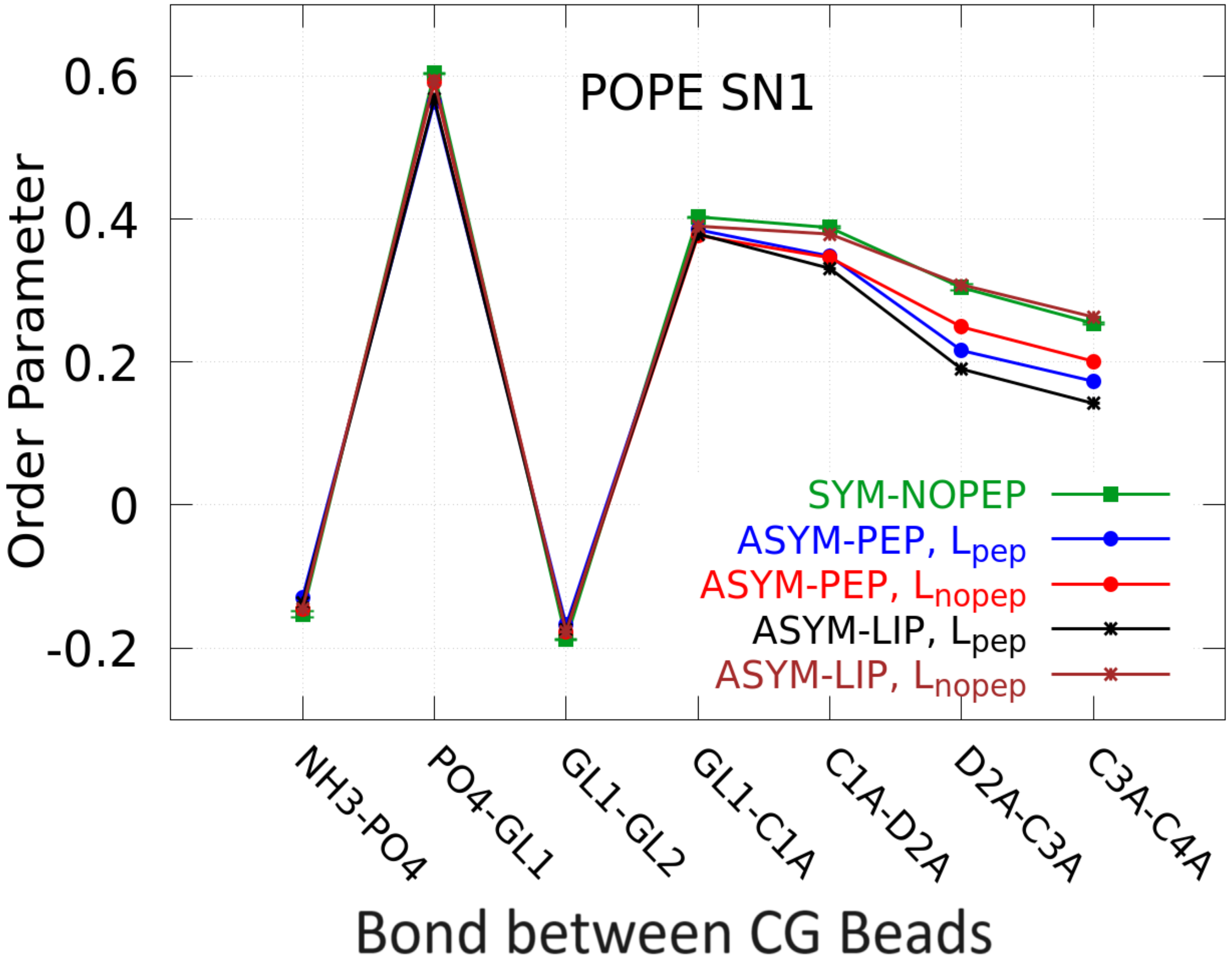

Figure S1: Lipid order parameter for SN1 tails of POPE from the upper and lower leaflets in the ASYM-PEP (with peptides on one of the leaflets), ASYM-LIP (the asymmetric membrane with peptides adsorbed on one leaflet and additional area-matching lipids in the opposite leaflet), and SYM-NOPEP (symmetric peptide-free) systems. L_{pep} indicates the leaflet with peptides and L_{nopep} the leaflet with no peptides in the ASYM-PEP and ASYM-LIP systems. Data for the SYM-NOPEP system are averaged over both leaflets and error bars show the standard deviation.

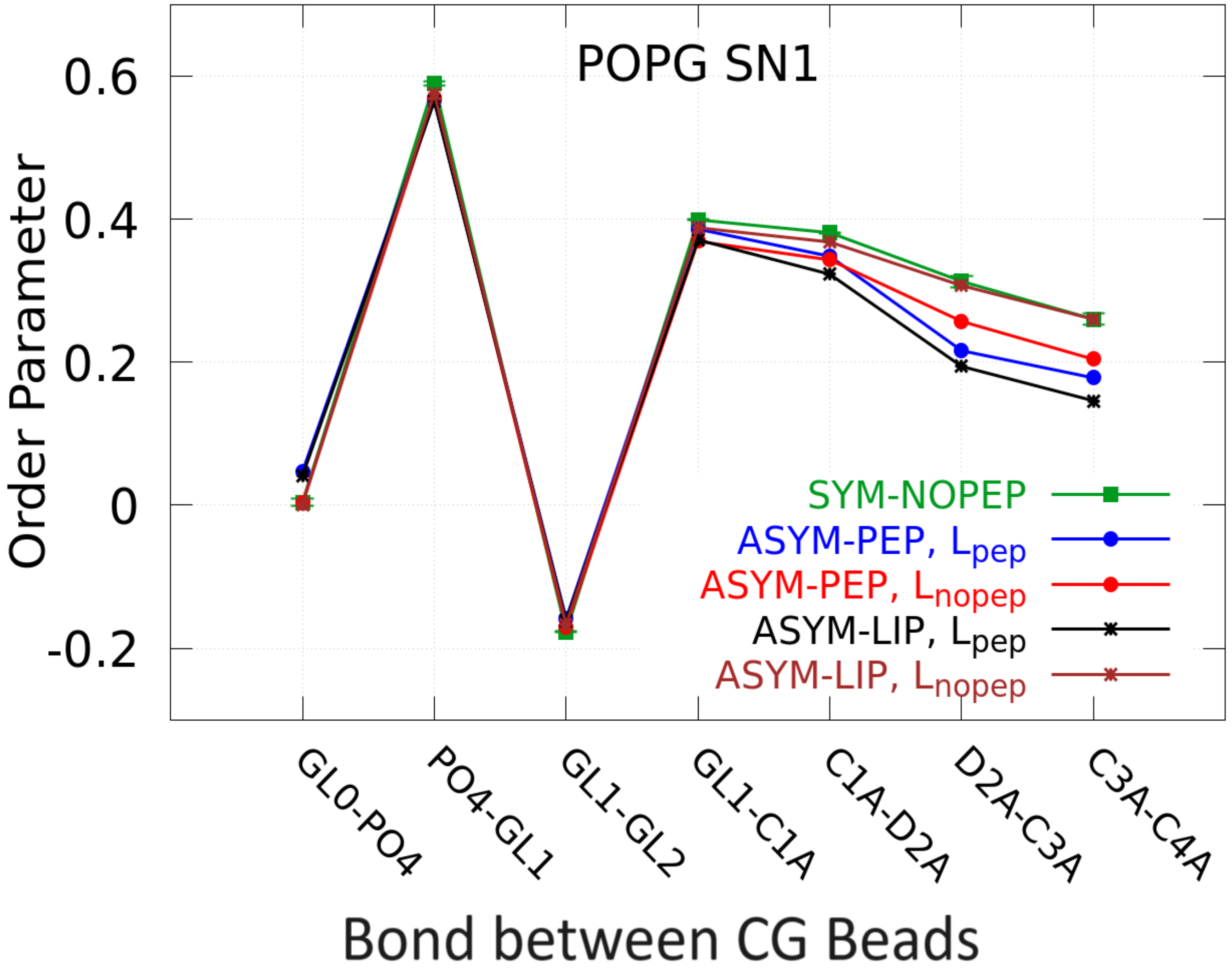

Figure S2: Lipid order parameter for SN1 tails of POPG from the upper and lower leaflets in the ASYM-PEP (with peptides on one of the leaflets), ASYM-LIP (the asymmetric membrane with peptides adsorbed on one leaflet and additional area-matching lipids in the opposite leaflet), and SYM-NOPEP (symmetric peptide-free) systems. L_{pep} indicates the leaflet with peptides and L_{nopep} the leaflet with no peptides in the ASYM-PEP and ASYM-LIP systems. Data for the SYM-NOPEP system are averaged over both leaflets and error bars show the standard deviation.

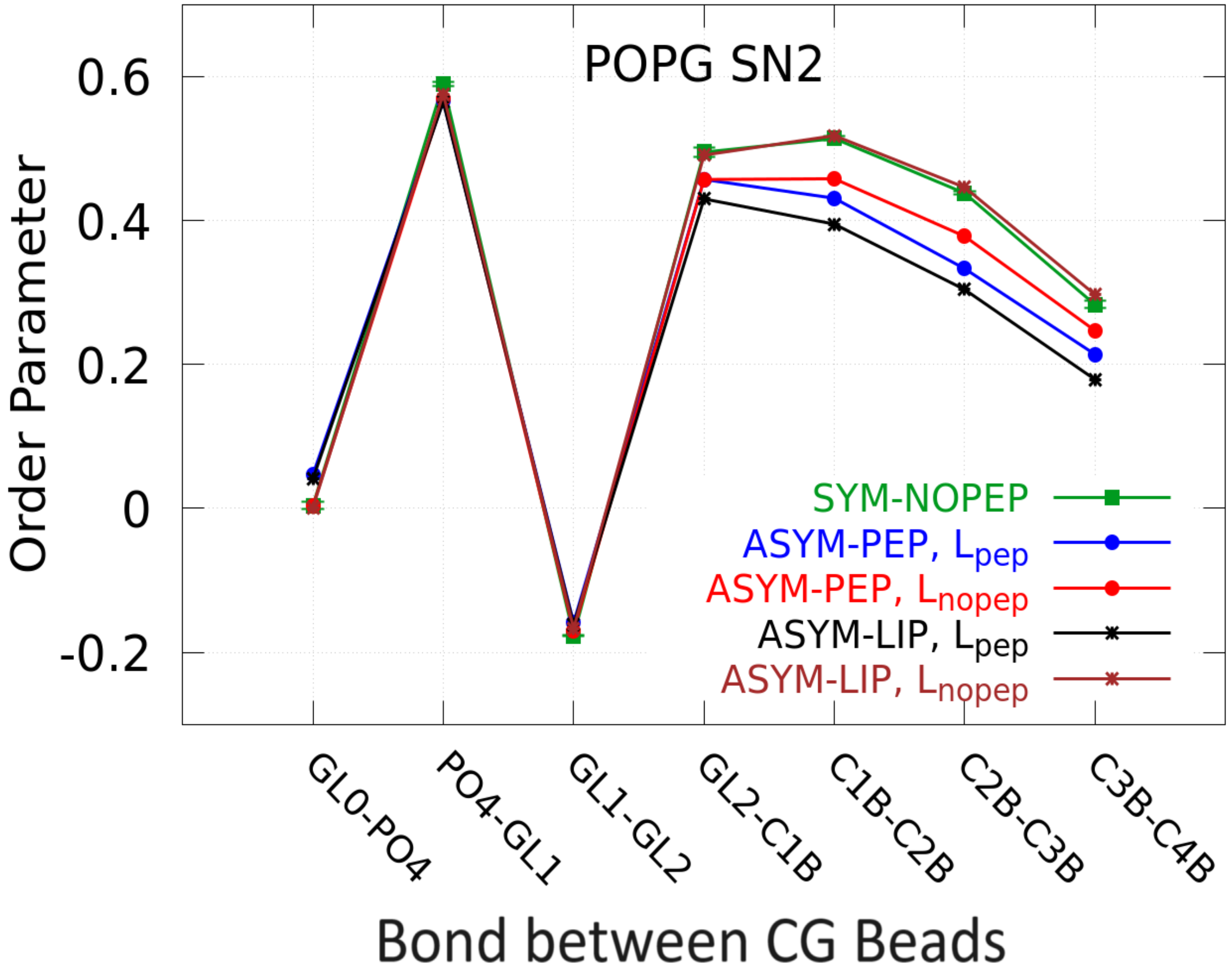

Figure S3: Lipid order parameter for SN2 tails of POPG from the upper and lower leaflets in the ASYM-PEP (with peptides on one of the leaflets), ASYM-LIP (the asymmetric membrane with peptides adsorbed on one leaflet and additional area-matching lipids in the opposite leaflet), and SYM-NOPEP (symmetric peptide-free) systems. L_{pep} indicates the leaflet with peptides and L_{nopep} the leaflet with no peptides in the ASYM-PEP and ASYM-LIP systems. Data for the SYM-NOPEP system are averaged over both leaflets and error bars show the standard deviation.

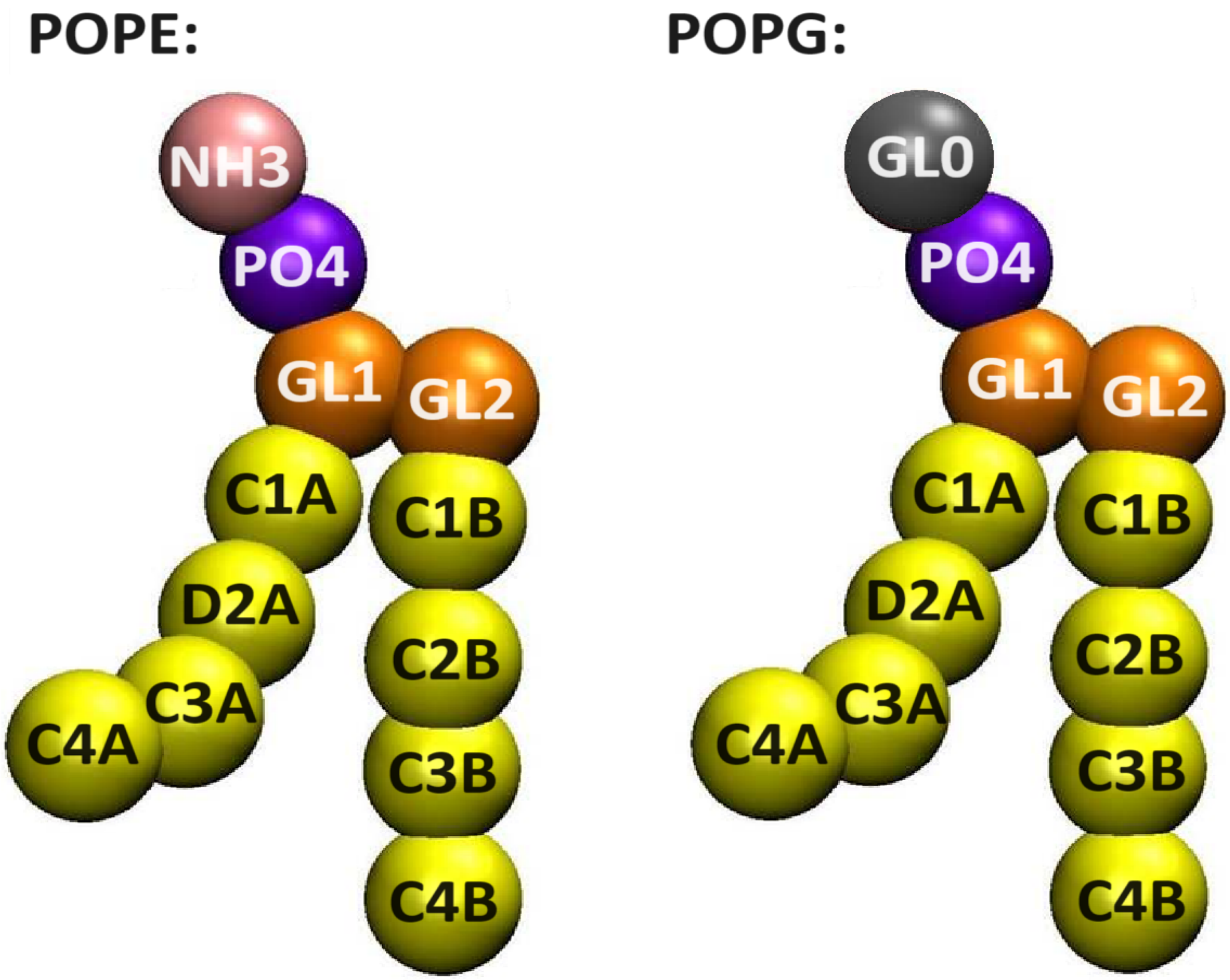

Figure S4: POPE and POPG representations at the coarse-grained level.

We calculated lipid order parameter using equation S5:

$$P_2 = \left\langle \frac{3\cos^2\theta - 1}{2} \right\rangle \tag{S5}$$

where P_2 is the order parameter, θ is the angle between the direction of the bond formed by two coarse-grained beads and the bilayer normal. P_2 value of 1 corresponds to perfect alignment (order), P_2 = -0.5 to perfect antialignment, and P_2 = 0 to a random orientation of bond with respect to the bilayer normal.
